# Reporting quality of clinical practice guidelines regarding gout and hyperuricemia according to the RIGHT checklist: systematic review

**DOI:** 10.1186/s13643-021-01645-1

**Published:** 2021-04-05

**Authors:** Can Wang, Xufei Luo, Maichao Li, Lingling Cui, Xinde Li, Lin Han, Xuefeng Wang, Wei Ren, Yuwei He, Wenyan Sun, Changgui Li, Yaolong Chen, Zhen Liu

**Affiliations:** 1 Gout Laboratory, The Affiliated Hospital of Qingdao University, No.16 Jiangsu Road, Qingdao, China; 2 School of Public Health, Lanzhou University, Lanzhou, China; 3 Institute of Metabolic Diseases, Qingdao University, Qingdao, China; 4 Institute of Health Data Science, Lanzhou University, Lanzhou, China; 5 Evidence-Based Medicine Center, School of Basic Medical Sciences, Lanzhou University, Lanzhou, China; 6 WHO Collaborating Centre for Guideline Implementation and Knowledge Translation, Lanzhou, China; 7 Guideline International Network Asia, Lanzhou, China; 8 Key Laboratory of Evidence Based Medicine and Knowledge Translation of Gansu Province, Lanzhou University, Lanzhou, China; 9 Lanzhou University GRADE Center, Lanzhou, China

**Keywords:** Clinical practice guideline, Gout, Hyperuricemia, RIGHT check list, Reporting quality

## Abstract

**Background:**

The Reporting Items for Practice Guidelines in Healthcare (RIGHT) checklist was used to assess the reporting quality of 2009–2019 clinical practice guidelines (CPGs) regarding gout and hyperuricemia, aimed to improve the reporting quality of future guidelines.

**Methods:**

We searched PubMed, the Chinese Biomedical Literature database, the Wanfang Database, and the China National Knowledge Infrastructure from January 2009 to June 2019 for guidelines regarding gout and hyperuricemia. We also searched the websites of guideline development organizations (the Guidelines International Network, the National Institute for Health and Clinical Excellence, the American College of Rheumatology, and the European League Against Rheumatism (EULAR)). Furthermore, supplementary guidelines reported in included articles were systematically searched, as well as Google Scholar.

**Results:**

Seventeen guidelines were included, of which one was in Chinese and 16 were in English. The mean reporting rate of the 35 items specified was 14.9 (42.5%); only five CPGs (29.4%) had a reporting rate >50%. Of the 35 items, three were very frequently reported. The reporting proportion of the seven domains (basic information, background, evidence, recommendations, review and quality assurance, funding and declaration and management of interests, and other information) were 64.7%, 36.8%, 50.6%, 42.9%, 8.82%, 33.8%, and 31.4%, respectively.

**Conclusion:**

The reporting quality of the present guidelines for gout and hyperuricemia is relatively poor. We suggest that the RIGHT reporting checklist should be used by CPG developers to ensure higher reporting quality of future guidelines.

**Supplementary Information:**

The online version contains supplementary material available at 10.1186/s13643-021-01645-1.

## Background

Gout is a group of heterogeneous diseases caused by long-term disturbance of purine metabolism, which results in a high serum uric acid concentration. The prevalence of gout is >1% in most developed countries, and with the recent improvement in living standards, the prevalence of gout is likely to increase [1]. This chronic disease is associated with substantial morbidity and mortality, making it a major global social and economic burden [2]. Furthermore, gout and hyperuricemia can induce and exacerbate metabolic diseases, such as hypertension, diabetes, and disorders of lipid metabolism; besides, they are also independent risk factors for stroke and myocardial infarction [3, 4]. Thus, gout and hyperuricemia have become common conditions that seriously affect human health.

With recent advances in pharmaceutical therapies, auxiliary diagnostic methods, and novel treatment approaches, a number of clinical practice guidelines (CPGs) have been developed to standardize the diagnosis and treatment of gout [5–9]. On the basis of evidence provided by systematic reviews, these CPGs have aimed to provide patients with the optimal medical treatment strategy [10]. Decisions regarding diagnosis and therapy are made on the basis of the CPGs, which standardize the behavior of clinicians, with the aims of improving clinical success and reducing cost [11]; therefore, the quality of CPGs is crucially important. There are two quality evaluation methods suitable for CPGs: the Appraisal of Guidelines for Research and Evaluation tool (AGREE) and the Reporting Items for Practice Guidelines in Healthcare (RIGHT) tool [12].

AGREE II [13] assesses the methodological rigor and transparency in which a CPG is developed and can be used to guide CPGs development, while RIGHT checklist [14] was developed in order to improve the reporting of practice guidelines. Because of the different purpose, appropriate instrument must be distinguished when addressing reporting or assessing methodological quality [12, 15]. A number of studies have been published that used AGREE II to assess the quality of the gout guidelines, which showed that the current guidelines are of poor quality [8, 16–18]. However, AGREE II does not separate the quality of the report contents from the quality of the methodology of CPGs; Yao et al. recommended RIGHT checklist providing detailed information that lacked in AGREE II [19], meaning that the reporting quality of gout and hyperuricemia guidelines has not been fully assessed [12]. Therefore, in the present study, we evaluated the reporting quality of gout and hyperuricemia CPGs using the RIGHT tool, to compensate previous study using AGREEII and permit the standardization reporting of future guidelines.

## Materials and methods

### Data source and search strategy

We searched PubMed, the Chinese Biomedical Literature database (CBM), the Wanfang Database, and the China National Knowledge Infrastructure (CNKI) from January 2009 to June 2019 for the relevant guidelines. We also searched the websites of the organizations responsible for guideline development: the Guidelines International Network (GIN), the National Institute for Health and Clinical Excellence (NICE), the American College of Rheumatology (ACR), and the European League Against Rheumatism (EULAR). Furthermore, supplementary guidelines reported in the included articles were systematically searched, and we also searched Google Scholar for additional material [8, 9, 16]; this project has been registered in OSFHOME (https://osf.io/z4evs/), and search strategy is listed in Supplementary file Table: 1.

### Study selection and data extraction

#### Inclusion criteria

“P” gout and/or hyperuricemia; “I” any intervention; “C” any comparator or comparison, no “key” CPG content is of interest; “A” 2009 to 2019, English or Chinese; “R” no recommendation is of interest [20].

#### Exclusion criteria

(1) Old versions of guidelines, if an updated version was available; (2) interpretations and translations of guidelines; (3) repeatedly published guidelines; and (4) guideline for which full text was still not available after contacting authors.

#### Data extraction

Two researchers (Zhen Liu and Can Wang) searched the database; all retrieved studies were screened using EndNote X8. After eliminating duplicates, the titles and abstracts were first screened according to the inclusion criteria, and the reasons for exclusion were recorded. Next, full text of the literature that met the inclusion criteria were screened again and determined whether the retrieved guidelines met the study criteria. Then, independent screening and cross-checking of the guidelines were carried out. Differences between reviewers were resolved through discussion or consultation with a third party (Yaolong Chen and his team).

### Reporting quality assessment

The RIGHT tool [14] (supplementary material) was used to evaluate the eligibility guidelines included in this study. The tool consists of 22 key items and 35 sub-items (Supplementary file Table: 2), which are divided into the following seven areas: basic information (items 1–4), background (items 5–9), evidence (items 10–12), recommendations (items 13–15), review and quality assurance (items 16–17), funding and declaration and management of interests (items 18–19), and other information (items 20–22). Each item was independently scored by two researchers, and most of the items were graded dichotomously, as “reported” (Y) or “unreported” (N). “Reported” meant that the relevant information was fully reported, whereas “unreported” meant the relevant information was unavailable. However, “partially reported” (P) was also used to indicate that the guideline contained only partial information, and when the guideline evaluation did not apply to the item, “not applicable” (NA) was recorded. We reported the results for each item as absolute quantities and percentages. For each item, we also reported the number and percentage of projects reported by each guideline. If the reporting proportion of the guidelines was <50%, the quality of the item was regarded as low [15]. The data were extracted and analyzed using Excel 2016 (Microsoft Corp., Redmond, WA, USA).

## Results

### Results of the selection

**Fig. 1:**
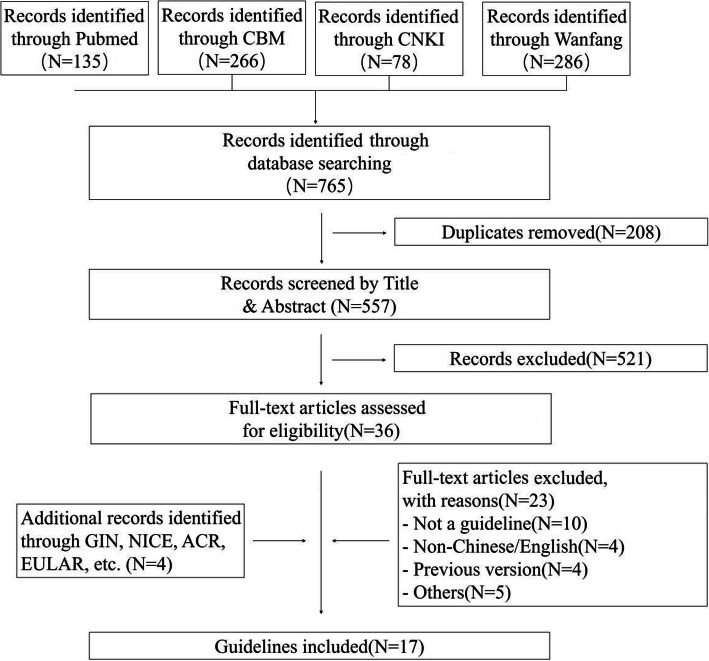
Literature selection flow chart

A total of 765 records were identified through database searching. Four other records were identified via the guideline development organization websites and Google Scholar. Of these, 17 guidelines that met the criteria were analyzed (Fig. 1) (Table 1) [21–39].

**Table 1:** Characteristics of included CPGs

Serial number	Title	Published date	Developer	Nation	Published journal
1	2018 updated European League Against Rheumatism evidence-based recommendations for the diagnosis of gout [21]	2019	European League Against Rheumatism.	Europe	Annals of the Rheumatic Diseases
2	Management of gout and hyperuricemia: multidisciplinary consensus in Taiwan [22]	2018	Astellas, Taiwan	Taiwan, China	Special editorial review
3	The British Society for Rheumatology guideline for the management of gout [23]	2017	British Society for Rheumatology Standards, Audit and Guidelines Working Group.	UK	Rheumatology
4	1. Diagnosis of acute gout: a clinical practice guideline from the American College of Physicians [24] 2. Management of acute and recurrent gout: A clinical practice guideline from the American College of Physicians [25]	2017	American College of Physicians	USA	Annals of Internal Medicine
5	[2016 China Gout Clinical Practice Guideline] [26]	2016	Chinese Rheumatology Association	China	Zhonghua Nei Ke Za Zhi
6	2016 updated EULAR evidence-based recommendations for the management of gout [27]	2016	European League Against Rheumatism	Europe	Clinical and epidemiological research
7	Treat-to-target (T2T) recommendations for gout [28]	2016	European League Against Rheumatism	Europe	Annals of the Rheumatic Diseases
8	Australian and New Zealand recommendations for the diagnosis and management of gout: integrating systematic literature review and expert opinion in the 3e initiative [29]	2015	APLAR, Asia Pacific League of Associations for Rheumatology	Australia and New Zealand	International Journal of Rheumatic Diseases
9	2015 gout classification criteria: an American College of Rheumatology/European League Against Rheumatism collaborative initiative [30]	2015	ACR/EULAR	USA and Europe	Annals of the Rheumatic Diseases
10	Portuguese recommendations for the diagnosis and management of gout [31]	2014	A panel of 78 international rheumatologists in 3e (Evidence, Expertise, Exchange) initiative	Portugal	Prática Clínica
11	Clinical practice guidelines for management of gout [32]	2013	Spanish Society of Rheumatology	Spain	GuipClinGot
12	Multinational evidence-based recommendations for the diagnosis and management of gout: integrating systematic literature review and expert opinion of a broad panel of rheumatologists in the 3e initiative [33]	2013	3e (Evidence, Expertise, Exchange) initiative/a panel of international rheumatologists	International	Annals of the Rheumatic Diseases
13	Italian Society of Rheumatology recommendations for the management of gout [34]	2013	Italian Society of Rheumatology	Italy	Reumatismo
14	Management of chronic gout in adults [35]	2012	University of Texas	USA	National Guideline Clearinghouse
15	1. 2012 American College of Rheumatology guidelines for management of gout. Part 1: systematic nonpharmacologic and pharmacologic therapeutic approaches to hyperuricemia [36] 2. 2012 American College of Rheumatology guidelines for management of gout. Part 2: therapy and anti-inflammatory prophylaxis of acute gouty arthritis [37]	2012	American College of Rheumatology	USA	Arthritis Care & Research
16	Japanese Guideline for the Management of Hyperuricemia and Gout: second edition [38]	2011	Tokyo Women’s Medical University	Japan	Nucleosides, Nucleotides and Nucleic Acids
17	Management of initial gout in adults [39]	2009	University of Texas	USA	National Guideline Clearinghouse

### Characteristics of the included CPGs

Of the 17 CPGs, one was in Chinese and 16 were in English. Nine were developed by European countries (52.9%), of which four were developed by EULAR (23.5%); six originated from the USA (35.3%), of which three were developed by ACR (17.6%); and three (17.6%) by universities (University of Texas, Tokyo Women’s Medical University, University of Texas). The remaining two (11.8%) were developed by the Evidence, Expertise, and Exchange initiative. The features of each are summarized (Table 1).

### Overall reporting quality

**Fig. 2:**
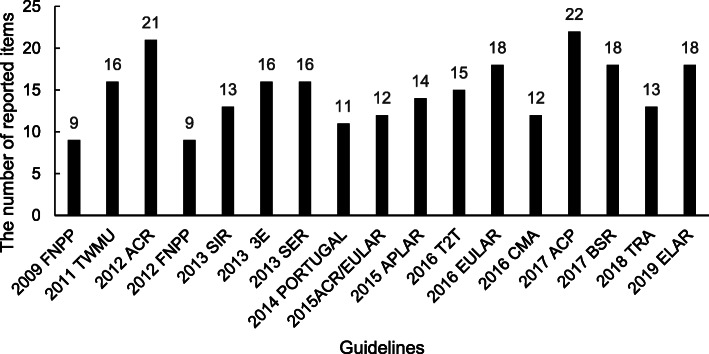
Number of reported items in each guideline

The mean reporting rate of the 35 items was 14.9 (42.5%), and it ranged from nine (25.7%) to 22 (62.9%) for the 17 CPGs (Fig. 2). Of the 17 CPGs, only five (29.4%) had a reporting rate >50%. The best one reported 62.9% of the items, while the poorest two reported <26% of the 35 items.

**Fig. 3:**
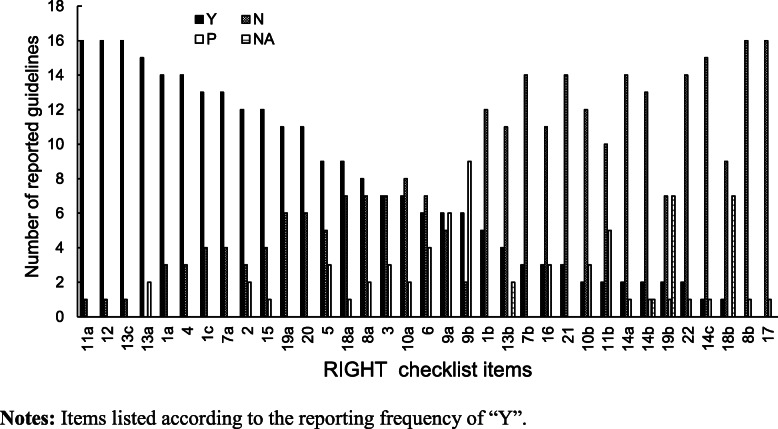
Number of guidelines reporting each item

Of the 35 items, the most frequently reported were #11a (indicate whether the guideline is based on new systematic reviews done specifically for this guideline or whether existing systematic reviews were used), #12 (describe the approach used to assess the certainty of the body of evidence), and #13c (indicate the strength of the recommendations and the certainty of the supporting evidence), which were reported in 16 (94.1%) guidelines. These were followed by #13a (provide clear, precise, and actionable recommendations), which was reported in 15 (88.2%) CPGs. Items #8b (describe the setting(s) for which the guideline is intended, such as primary care, low- and middle-income countries, or inpatient facilities) and #17 (indicate whether the guideline was subjected to a quality assurance process. If yes, describe the process) were reported in none of the CPGs (Fig. 3) (Table 2).

**Table 2:** Quality of the guidelines, according to the reporting of each item on the RIGHT checklist

		17	16	15	14	13	12	11	10	9	8	7	6	5	4	3	2	1	
		2009 FNPP	2011 TWMU	2012 ACR	2012 FNPP	2013 SIR	2013 3E	2013 SER	2014 Portugal	2015ACR/EULAR	2015 APLAR	2016 T2T	2016 EULAR	2016 CMA	2017 ACP	2017 BSR	2018 TRA	2019 ELAR	Total
Basic information	1a	N	Y	Y	N	Y	Y	Y	Y	N	Y	Y	Y	Y	Y	Y	Y	Y	14
	1b	N	N	Y	N	N	N	N	N	Y	N	N	Y	Y	N	N	N	Y	5
	1c	Y	Y	Y	Y	Y	Y	Y	Y	Y	Y	N	Y	N	Y	N	N	Y	13
	2	P	Y	Y	P	Y	Y	N	Y	Y	Y	Y	Y	N	Y	N	Y	Y	12
	3	N	N	P	N	P	Y	Y	Y	N	N	Y	P	N	Y	N	Y	Y	7
	4	N	Y	Y	N	Y	Y	N	Y	Y	Y	Y	Y	Y	Y	Y	Y	Y	14
Total		1	4	5	1	4	5	3	5	4	4	4	5	3	5	2	4	6	65
Background	5	N	N	Y	N	Y	P	Y	N	Y	Y	N	P	Y	Y	Y	Y	P	9
	6	N	Y	Y	Y	N	N	Y	P	N	P	P	N	N	Y	Y	N	P	6
	7a	Y	Y	Y	Y	N	N	N	N	Y	Y	Y	Y	Y	Y	Y	Y	Y	13
	7b	N	Y	N	N	N	N	N	N	N	N	N	Y	N	N	Y	N	N	3
	8a	Y	N	Y	Y	N	N	Y	N	N	N	N	P	Y	Y	Y	Y	P	8
	8b	N	N	N	N	N	N	N	N	N	N	N	N	N	N	N	N	P	0
	9a	N	N	Y	N	N	P	Y	P	N	Y	P	Y	P	Y	P	P	Y	6
	9b	N	N	P	P	P	P	Y	P	P	P	Y	Y	Y	Y	P	P	Y	6
Total		2	3	5	3	1	0	5	0	2	3	2	4	4	6	5	3	3	51
Evidence	10a	N	N	Y	N	Y	Y	P	Y	N	Y	N	P	N	N	Y	Y	N	7
	10b	N	N	N	N	Y	N	N	N	N	N	Y	N	P	P	P	N	N	2
	11a	Y	Y	Y	Y	Y	Y	Y	Y	Y	Y	Y	Y	Y	Y	Y	N	Y	16
	11b	P	N	N	N	N	Y	P	N	N	N	N	N	P	Y	P	P	N	2
	12	Y	Y	Y	Y	Y	Y	Y	Y	N	Y	Y	Y	Y	Y	Y	Y	Y	16
Total		2	2	3	2	4	4	2	3	1	3	3	2	2	3	3	2	2	43
Recommendations	13a	P	Y	Y	P	Y	Y	Y	Y	Y	Y	Y	Y	Y	Y	Y	Y	Y	15
	13b	N	Y	N	N	N	N	N	N	N	N	N	Y	N	Y	Y	NA	NA	4
	13c	Y	Y	Y	Y	Y	Y	Y	Y	N	Y	Y	Y	Y	Y	Y	Y	Y	16
	14a	N	Y	N	N	N	N	N	N	N	Y	N	N	N	N	N	N	P	2
	14b	N	N	N	N	Y	N	N	N	N	N	N	N	NA	N	Y	N	P	2
	14c	N	N	N	N	Y	N	N	N	N	N	N	N	N	N	P	N	N	1
	15	N	Y	Y	N	N	Y	Y	Y	Y	Y	Y	Y	P	N	Y	Y	Y	12
Total		1	5	3	1	4	3	3	3	2	4	3	4	2	3	5	3	3	52
Review and quality assurance	16	Y	Y	N	N	N	N	N	N	N	N	P	P	N	Y	N	N	P	3
	17	N	N	N	N	N	N	N	N	N	N	N	N	N	N	P	N	N	0
Total		1	1	0	0	0	0	0	0	0	0	0	0	0	1	0	0	0	3
Funding and declaration and management of interests	18a	Y	N	Y	Y	N	Y	Y	N	Y	N	Y	N	N	Y	N	P	Y	9
	18b	N	NA	N	N	NA	N	Y	NA	N	NA	N	NA	NA	N	NA	N	N	1
	19a	N	N	Y	N	N	Y	Y	N	Y	N	Y	Y	Y	Y	Y	Y	Y	11
	19b	NA	NA	N	NA	NA	N	N	NA	N	N	N	P	NA	Y	NA	N	Y	2
Total		1	0	2	1	0	2	3	0	2	0	2	1	1	3	1	1	3	23
Other information	20	Y	Y	Y	Y	N	Y	N	N	Y	N	Y	Y	N	Y	Y	N	Y	11
	21	N	N	Y	N	N	N	N	N	N	N	N	Y	N	N	Y	N	N	3
	22	N	N	Y	N	N	Y	N	N	N	P	N	N	N	N	N	N	N	2
Total		1	1	3	1	0	2	0	0	1	0	1	2	0	1	2	0	1	16
		9	16	21	9	13	16	16	11	12	14	15	18	12	22	18	13	18	253

According to the RIGHT tool, the reporting rates of the seven domains (basic information, background, evidence, recommendations, review and quality assurance, statements and management of funds and interests, and other information) were 64.7%, 36.8%, 50.6%, 42.9%, 8.82%, 33.8%, and 31.4%, respectively.

### Subgroup analysis

According to the geographical regions of the sponsors or participants, nine CPGs were participated in or initiated by European countries, and 6 CPGs originated from American countries. Firstly, the mean number of reporting items of the 9 CPGs developed by European countries was 15.2, and only 3 CPGs had a compliance rate of >50% (33.3%); four of these 9 CPGs were EULAR-generated; the mean number of reporting items in these 4 CPGs was 15.8. Secondly, as for the six CPGs developed in the USA, the mean number of reporting item was 14.8. Two of these 6 CPGs were prepared by the ACR, with a mean number of reporting items of 16.5, and two were published on NGC (National Guideline Clearinghouse), reported only nine items. Twenty-two items were reported in the American College of Physicians (ACP) CPG, which was the guideline with the largest number of reporting items among the 17 included in the analysis.

Eight CPGs were published between 2009 and 2014, which had a mean of 13.9 reporting items, and only one (12.5%) guideline had a compliance rate of more than 50%. Between 2015 and 2019, a total of nine CPGs were published, with a mean of 15.8 items being reported, but only four (44.4%) had a compliance rate of >50%.

### Analysis of each section of the RIGHT checklist

#### Basic information

With regard to the basic information, most of the CPGs could be judged by their title (82.4%), and the contact information for at least one author could be found (82.4%). The classification of the guidelines could be easily discerned in 76.5% of the CPGs, and a summary was presented in 70.5%. However, only 29.4% of the CPGs reported a publication date. Forty-one-point-two percent of the CPGs defined new terms and gave corresponding acronyms.

#### Background

The 17 CPGs did not comprehensively describe the background. More than half reported the epidemiology of gout (52.9%). Six (35.3%) CPGs reported the overall objectives of the guidelines and the specific objectives they were designed to achieve. Most of the guidelines described the main target population (76.5%), but only 17.6% described the subgroups that should be considered. Fewer than half (47.1%) of the guidelines described the key users and other potential users of the guidelines, and none described the specific target environment. Six (35.3%) CPGs listed all the contributors and their roles, and six (35.3%) gave the titles and affiliations of all the participants in their development, but only four (23.5%) contained both sets of information.

#### Evidence

Seven (41.2%) of the CPGs described the key findings on which the recommendations were based, but only two described the selection and classification of outcomes, and only one reported both. Sixteen (94.1%) guidelines described whether the systematic reviews on which the guidelines were based had been recently completed, but only two (11.8%) presented references, described how they were retrieved and evaluated, and stated whether they had been updated. Only one CPG did not describe the method of evaluation and grading of the quality of evidence.

#### Recommendations

Most of the CPGs (15, 80.2%) provided clear, accurate, and enforceable recommendations. Sixteen (94.1%) CPGs described the strength of the recommendation and the quality of the evidence supporting it, but only four provided recommendations for subgroups. Four CPGs took into account the preferences and values of the target group, two (11.8%) took into account cost and resource utilization, and two (11.8%) took into account fairness, feasibility, and acceptability, when creating their recommendations. Most of the CPGs (15, 88.2%) described the decision-making process and the methods used by the working group that created the guideline.

#### Review and quality assurance

Only three (17.6%) CPGs described whether they had been sent for review or not, and none described the level of supervision involved.

#### Funding and declaration and management of interests

More than half of the CPGs (9, 52.9%) described the sources of funding at each stage of their development, but only one described the role of the sponsor in the different stages of guideline formulation, as well as in the dissemination and implementation of the recommendations. Similarly, most of the CPGs (11, 64.7%) described the types of conflict of interest associated, but only two (11.8%) described the evaluation and management of these conflicts of interest and how users of the guidelines could obtain this information.

#### Other information

Eleven (64.7%) CPGs described where the guidelines, corresponding attachments, and other related documents could be obtained. However, only three (17.6%) articles described the differences between current practice and that recommended by research evidence, and/or provided recommendations for future research. In addition, only two CPGs described all the limitations associated with the formulation of the guidelines and their possible impact on the effectiveness of the recommendations.

## Discussion

The quality of CPGs about gout and hyperuricemia were assessed before with AGREE II [5], whereas AGREE II and RIGHT checklist had unique items by themselves [19], the RIGHT checklist added new and detailed items that AGREE II lacked. We assessed the reporting quality of CPGs in the field of gout and hyperuricemia, using the RIGHT checklist in our study, to help guideline developers better standardizing the reporting quality of future CPGs. Furthermore, it also could help healthcare professionals better understand and implement the contents of such CPGs [12]. We used the RIGHT checklist to evaluate 17 guidelines for gout and hyperuricemia of various types that had been prepared in different geographical regions over the past 10 years, and found that they varied in quality. Of the 17 CPGs, only five reported >50% of the items in the RIGHT tool, considering our team’s consensus: If reporting proportion of guidelines <50%, the quality was regarded as low [15], suggesting that most CPGs were of low reporting quality. This finding is similar to that made previously using the AGREEIItool [16].

Among the seven sections of the RIGHT tool, the reporting rate of basic information was the highest (64.7%), followed by evidence (50.6%), but the reporting rate of the other sections was <50%. The most poorly reported section was the declaration and management of funds and interests, with only 8.8% of CPGs reporting these items. This suggests that the guideline creators are generally good at including basic information, but that review and quality assurance are easily ignored.

Most of the CPGs were written by European and American organizations, and the overall completeness of the guidelines was slightly better in Europe than that in the USA. However, the scores for the CPGs written by the ACR and ACP, which are influential guideline-writing organizations in the USA, were higher than those for EULAR-generated CPGs. This shows that there are differences in reporting quality of the CPGs written by different organizations in different geographical regions. With regard to the relationship between publication date and score, the compilation of guidelines has greatly improved in recent years. The guideline writers have aimed to correct the deficits of the previous CPGs with regard to the practical applications of the guidelines, but the emergence of guideline-based systematic reviews in recent years have also made them consider writing their guidelines in accordance with certain norms.

### Analysis of the reporting quality of guidelines regarding gout and hyperuricemia

#### Basic information

Most of the CPGs had a high reporting rate for basic information, but there were still some in which the year of publication was not clear and there was ambiguity in the definitions of acronyms. These issues are very important for researchers and practitioners who wish to know how up-to-date guidelines are, and it is necessary to interpret vague terms and acronyms for more accurate understanding of the guidelines.

#### Background

In the background of the CPGs, the writers usually described the main target groups for the guidelines and discussed the epidemiology of the problems described. However, the other items were frequently not well reported. For example, the inclusion of 8a (the main users of the guide should be described) and 8b (the specific environment for which the guide should be described) demonstrate that the application of the guidelines will vary for different users and different environments. For example, for low- and middle-income people, it is necessary to consider the economic benefits [15]. Therefore, the background of the guidelines should be described in detail.

#### Evidence

About half of the items in the evidence section were reported, and items 10b (the methodology for the selection and classification of outcomes should be described) and 11b (if guide makers use published systematic reviews, references should be given and describe how they were retrieved and evaluated) had low reporting rates. Items 10b and 11b can greatly help researchers and practitioners to understand the evidence and assess the accuracy of the guidelines. Furthermore, they are important for peer review because they permit shortcomings in the CPGs to be found and remedied. Items 10b and 11b can also be supplemented as new evidence accumulates over time.

#### Recommendations

Most of the guidelines did not make recommendations for subgroups, suggesting that the writers did not regard subgroups as important. Most writers did not consider items 14a (whether to consider the preferences and values of the target population), 14b (whether to consider cost and resource utilization), or 14c (whether to consider fairness, feasibility, and acceptability), or they once considered these issues but did not included them in the CPGs. This means that healthcare professionals cannot easily adapt the CPGs according to different clinical situations.

#### Review and quality assurance

Items in this section were described in only a small number of CPGs, such that the reporting rate was the lowest for all the sections. Some of the guidelines may have been independently reviewed and quality-controlled, but the absence of such information is likely to make guide users and peer reviewers doubt the quality of the guidelines.

#### Funding and declaration and management of interests

The RIGHT checklist showed that the quality of the published CPGs was low with regard to this aspect, suggesting that the writers did not pay enough attention to it. A lack of information of this section may lead to the inference that the recommendations of these CPGs could have been influenced by multiple interested parties, which would lower the credibility of the guidelines, especially with regard to specific treatment recommendations.

#### Other information

Most of the CPGs failed to differentiate evidence obtained from practice and research or to provide recommendations for future research (or both). They also failed to describe the limitations in the formulation of the CPGs and the possible impact of these limitations on the effectiveness of the recommendations. Such content could have provided a reference for guide users to appropriately use the recommendations and provided guidance for future updates and other researches.

### Strengths and limitation

#### Strengths

This is the first study using the RIGHT tool to evaluate the reporting quality of CPGs in the field of gout and hyperuricemia. In addition, the methods of systematic retrieval, screening, and evaluation were adopted, and the quality of the outcome measurements was strictly controlled.

#### Limitations

First, only CPGs written in Chinese and English were included; this might cause language bias. Second, only CPGs published in journals and online regarding gout and hyperuricemia were included; CPGs published in the form of books or government documents were not analyzed.

## Conclusion

In conclusion, most of the current CPGs in the field of gout and hyperuricemia had relatively low scores, when evaluated by using the RIGHT checklist. CPGs with poor reporting quality might mislead users and lead to wrong diagnosis and/or treatment, resulting in a waste of medical resources and/or delay of the disease. We suggest organizations that participate in reporting of CPGs regarding gout and hyperuricemia to use RIGHT tool, so as to improve standardization of reporting, making the reporting clear, complete, and transparent.

## Supplementary Information

**Additional file 1.**

## Abbreviations

RIGHT: Reporting Items for Practice Guidelines in Healthcare
CPGs: Clinical Practice Guidelines
EULAR: European League Against Rheumatism
AGREE: Appraisal of Guidelines for Research and Evaluation tool
CBM: Chinese Biomedical Literature database
CNKI: China National Knowledge Infrastructure
GIN: Guidelines International Network
NICE: National Institute for Health and Clinical Excellence
ACR: American College of Rheumatology
NGC: National Guideline Clearinghouse
ACP: American College of Physicians

## References

[1] Xia Y, Wu Q, Wang H, Zhang S, Jiang Y, Gong T, et al. Global, regional and national burden of gout, 1990-2017: a systematic analysis of the Global Burden of Disease Study. Rheumatology (Oxford). 2020;59(7):1529–38. 10.1093/rheumatology/kez476. 10.1093/rheumatology/kez476 31624843

[2] Rai SK, Burns LC, de Vera MA, Haji A, Giustini D, Choi HK (2015). The economic burden of gout: a systematic review. Semin Arthritis Rheum.

[3] Shiozawa A, Szabo SM, Bolzani A, Cheung A, Choi HK (2017). Serum uric acid and the risk of incident and recurrent gout: a systematic review. J Rheumatol.

[4] MacFarlane LA, Kim SC (2014). Gout: a review of nonmodifiable and modifiable risk factors. Rheum Dis Clin North Am.

[5] Wang D, Yu Y, Chen Y, Yang N, Zhang H, Wang C, et al. Assessing the quality of global clinical practice guidelines on gout using AGREE II instrument. J Clin Rheumatol. 2020;26(2):54–9. 10.1097/RHU.0000000000000921. 10.1097/RHU.0000000000000921 32073515

[6] Yu Y, et al. Recommendations in clinical practice guidelines on gout: systematic review and consistency analysis. Clin Exp Rheumatol. 2020. 31969230

[7] Ughi N, Prevete I, Ramonda R, Cavagna L, Filippou G, Manara M, et al. The Italian Society of Rheumatology clinical practice guidelines for the diagnosis and management of gout. Reumatismo. 2019;71(S1):50–79. 10.4081/reumatismo.2019.1176. 10.4081/reumatismo.2019.1176 31948193

[8] Li Q, Li X, Wang J, Liu H, Kwong JS, Chen H, et al. Diagnosis and treatment for hyperuricemia and gout: a systematic review of clinical practice guidelines and consensus statements. BMJ Open. 2019;9(8):e026677. 10.1136/bmjopen-2018-026677. 10.1136/bmjopen-2018-026677 PMC6720466 31446403

[9] Yang N, Yu Y, Zhang A, Estill J, Wang X, Zheng M, et al. Reporting, presentation and wording of recommendations in clinical practice guideline for gout: a systematic analysis. BMJ Open. 2019;9(1):e024315. 10.1136/bmjopen-2018-024315. 10.1136/bmjopen-2018-024315 PMC6352818 30700479

[10] Institute of Medicine (US) Committee on Standards for Developing Trustworthy Clinical Practice Guidelines. Clinical practice guidelines we can trust. Washington, DC: National Academies Press; 2011.

[11] Grilli R, Magrini N, Penna A, Mura G, Liberati A (2000). Practice guidelines developed by specialty societies: the need for a critical appraisal. Lancet.

[12] Chen Y, Yang K, Marušic A, Qaseem A, Meerpohl JJ, Flottorp S, et al. A reporting tool for practice guidelines in health care: the RIGHT statement. Ann Intern Med. 2017;166(2):128–32. 10.7326/M16-1565. 10.7326/M16-1565 27893062

[13] Brouwers MC, Kho ME, Browman GP, Burgers JS, Cluzeau F, Feder G, et al. AGREE II: advancing guideline development, reporting and evaluation in health care. CMAJ. 2010;182(18):E839–42. 10.1503/cmaj.090449. 10.1503/cmaj.090449 PMC3001530 20603348

[14] Chen Y, Yang K, Marušić A, Qaseem A, Meerpohl JJ, Flottorp S, et al. A reporting tool for practice guidelines in health care: the RIGHT statement. Z Evid Fortbild Qual Gesundhwes. 2017;127-128:3–10. 10.1016/j.zefq.2017.10.008. 10.1016/j.zefq.2017.10.008 29128430

[15] Xiao Y, Jiang L, Tong Y, Luo X, He J, Liu L, et al. Evaluation of the quality of guidelines for assisted reproductive technology using the RIGHT checklist: a cross-sectional study. Eur J Obstet Gynecol Reprod Biol. 2019;241:42–8. 10.1016/j.ejogrb.2019.07.039. 10.1016/j.ejogrb.2019.07.039 31419695

[16] Wang D, et al. Assessing the quality of global clinical practice guidelines on gout using AGREE II instrument. J Clin Rheumatol. 2018. 10.1097/RHU.0000000000000921 32073515

[17] Collaboration, A (2003). Development and validation of an international appraisal instrument for assessing the quality of clinical practice guidelines: the AGREE project. Qual Saf Health Care.

[18] Oxman AD, Schunemann HJ, Fretheim A (2006). Improving the use of research evidence in guideline development: 16. Evaluation. Health Res Policy Syst.

[19] Yao X, Ma J, Wang Q, Kanters D, Ali MU, Florez ID (2020). A comparison of AGREE and RIGHT: which clinical practice guideline reporting checklist should be followed by guideline developers?. J Gen Intern Med.

[20] Johnston A, Kelly SE, Hsieh SC, Skidmore B, Wells GA (2019). Systematic reviews of clinical practice guidelines: a methodological guide. J Clin Epidemiol.

[21] Richette P, Doherty M, Pascual E, Barskova V, Becce F, Castaneda J, et al. 2018 updated European League Against Rheumatism evidence-based recommendations for the diagnosis of gout. Ann Rheum Dis. 2020;79(1):31–8. 10.1136/annrheumdis-2019-215315. 10.1136/annrheumdis-2019-215315 31167758

[22] Yu KH, Chen DY, Chen JH, Chen SY, Chen SM, Cheng TT, et al. Management of gout and hyperuricemia: multidisciplinary consensus in Taiwan. Int J Rheum Dis. 2018;21(4):772–87. 10.1111/1756-185X.13266. 10.1111/1756-185X.13266 29363262

[23] Hui M, Carr A, Cameron S, Davenport G, Doherty M, Forrester H, et al. The British Society for Rheumatology guideline for the management of gout. Rheumatology (Oxford). 2017;56(7):1246. 10.1093/rheumatology/kex250. 10.1093/rheumatology/kex250 28605531

[24] Qaseem A, McLean RM, Starkey M, Forciea MA, for the Clinical Guidelines Committee of the American College of Physicians (2017). Diagnosis of acute gout: a clinical practice guideline from the American College of Physicians. Ann Intern Med.

[25] Qaseem A, Harris RP, Forciea MA, for the Clinical Guidelines Committee of the American College of Physicians (2017). Management of acute and recurrent gout: a clinical practice guideline from the American College of Physicians. Ann Intern Med.

[26] Chinese Rheumatology A (2016). China gout clinical practice guideline. Zhonghua Nei Ke Za Zhi.

[27] Richette P, Doherty M, Pascual E, Barskova V, Becce F, Castañeda-Sanabria J, et al. 2016 updated EULAR evidence-based recommendations for the management of gout. Ann Rheum Dis. 2017;76(1):29–42. 10.1136/annrheumdis-2016-209707. 10.1136/annrheumdis-2016-209707 27457514

[28] Kiltz U, Smolen J, Bardin T, Cohen Solal A, Dalbeth N, Doherty M, et al. Treat-to-target (T2T) recommendations for gout. Ann Rheum Dis. 2017;76(4):632–8. 10.1136/annrheumdis-2016-209467. 10.1136/annrheumdis-2016-209467 27658678

[29] Graf SW, Whittle SL, Wechalekar MD, Moi JHY, Barrett C, Hill CL, et al. Australian and New Zealand recommendations for the diagnosis and management of gout: integrating systematic literature review and expert opinion in the 3e initiative. Int J Rheum Dis. 2015;18(3):341–51. 10.1111/1756-185X.12557. 10.1111/1756-185X.12557 25884565

[30] Neogi T, Jansen TLTA, Dalbeth N, Fransen J, Schumacher HR, Berendsen D, et al. 2015 gout classification criteria: an American College of Rheumatology/European League Against Rheumatism collaborative initiative. Ann Rheum Dis. 2015;74(10):1789–98. 10.1136/annrheumdis-2015-208237. 10.1136/annrheumdis-2015-208237 PMC4602275 26359487

[31] Araujo F (2014). Portuguese recommendations for the diagnosis and management of gout. Acta Reumatol Port.

[32] Spanish Society of Rheumatology. Clinical practice guidelines for management of gout. 2013. Available from: https://www.guidelinecentral.com/summaries/clinical-practice-guidelines-for-management-of-gout/#section-society.

[33] Sivera F, Andrés M, Carmona L, Kydd ASR, Moi J, Seth R, et al. Multinational evidence-based recommendations for the diagnosis and management of gout: integrating systematic literature review and expert opinion of a broad panel of rheumatologists in the 3e initiative. Ann Rheum Dis. 2014;73(2):328–35. 10.1136/annrheumdis-2013-203325. 10.1136/annrheumdis-2013-203325 PMC3913257 23868909

[34] Manara M, Bortoluzzi A, Favero M, Prevete I, Scirè CA, Bianchi G, et al. Italian Society of Rheumatology recommendations for the management of gout. Reumatismo. 2013;65(1):4–21. 10.4081/reumatismo.2013.4. 10.4081/reumatismo.2013.4 23550256

[35] The University of Texas at Austin, S.o.N., Family Nurse Practitioner Program, , Management of chronic gout in adults. Available from: https://www.ahrq.gov/gam/index.html., 2012.

[36] Khanna D, Fitzgerald JD, Khanna PP, Bae S, Singh MK, Neogi T, et al. American College of Rheumatology guidelines for management of gout. Part 1: systematic nonpharmacologic and pharmacologic therapeutic approaches to hyperuricemia. Arthritis Care Res (Hoboken), 2012. 2012;64(10):1431–46. 10.1002/acr.21772. 10.1002/acr.21772 PMC3683400 23024028

[37] Khanna D, Khanna PP, Fitzgerald JD, Singh MK, Bae S, Neogi T, et al. American College of Rheumatology guidelines for management of gout. Part 2: therapy and antiinflammatory prophylaxis of acute gouty arthritis. Arthritis Care Res (Hoboken), 2012. 2012;64(10):1447–61. 10.1002/acr.21773. 10.1002/acr.21773 PMC3662546 23024029

[38] Yamanaka H, Japanese Society G (2011). of, and M. Nucleic Acid, Japanese guideline for the management of hyperuricemia and gout: second edition. Nucleosides Nucleotides Nucleic Acids.

[39] The University of Texas at Austin, School of Nursing, Family Nurse Practitioner Program, Management of initial gout in adults. 2009. [cited September 12 2018]. Available from: https://www.ahrq.gov/gam/index.html.

